# ‘Leaving no one behind’: a scoping review on the provision of sexual and reproductive health care to nomadic populations

**DOI:** 10.1186/s12905-019-0849-4

**Published:** 2019-12-16

**Authors:** Moazzam Ali, Joanna Paula Cordero, Faria Khan, Rachel Folz

**Affiliations:** Department of Reproductive Health and Research, World Health Organization, Avenue Appia 20, CH-1211 Geneva, Switzerland

**Keywords:** Nomad, Pastoralist, Sexual and reproductive health, Family planning, Health services

## Abstract

**Backgrund:**

Marginalized groups, such as nomadic populations across the world, have perhaps the least access to modern reproductive health (RH) services. This scoping review aims to identify barriers to access to RH services faced by nomadic populations from the existing literature and to highlight possible opportunities to address them.

**Methods:**

Key databases, including PubMed, Popline, Google Scholar, and Google Advanced were searched for relevant articles published between 2002 and 2019. A total 344 articles were identified through database online searches, and 31 were included in the review.

**Results:**

Nomadic people face complex barriers to healthcare access that can be broadly categorized as external (geographic isolation, socio-cultural dynamics, logistical and political factors) and internal (lifestyle, norms and practices, perceptions) factors. To effectively address the needs of nomadic populations, RH services must be available, accessible and acceptable through tailored and culturally sensitive approaches. A combination of fixed and mobile services has proven effective among mobile populations. Low awareness of modern RH services and their benefits is a major barrier to utilization. Partnership with communities through leveraging existing structures, networks and decision-making patterns can ensure that the programmes are effectively implemented.

**Conclusion:**

Further research is needed to better understand and address the RH needs of nomadic populations. Though existing evidence is limited, opportunities do exist and should be explored. Raising awareness and sensitization training among health providers about the specific needs of nomads is important. Improved education and access to information about the benefits of modern RH care among nomadic communities is needed. Ensuring community participation through involvement of nomadic women and girls, community leaders, male partners, and trained traditional birth attendants are key facilitators in reaching nomads. However, participatory programmes also need to be recognized and supported by governments and existing health systems.

## Background

Despite the great progress towards improving reproductive health (RH) for global populations, outcomes such as maternal morbidity and mortality and a global unmet need for contraception remain large concerns in the international public health community [1, 2]. The Sustainable Development Goals (SDG) for 2030, developed by the United Nations highlight the need for a reduction of maternal mortality and of unmet contraceptive needs as important targets to improving health and wellbeing of the global community. The SDGs also emphasize the importance of gender equality, and improved experiences of women and girls internationally [3]. To reach these important goals, it is essential that we focus on populations who may be marginalized, and whose outcomes lag behind those of the general population.

For that reason, it is important to focus on the health and wellbeing of nomadic populations, whose access to healthcare, including RH services, is much more limited than that of the general population [4]. Nomads are defined broadly as people whose existence is marked by mobility as they move from place to place, generally in traditional routes, in search of resources and food. Our paper focuses on pastoralist nomads, whose trajectories largely rely on grazing grounds for livestock. Pastoralists are defined as groups that move their homes according to grazing opportunities for their livestock and make up a large proportion of the global nomadic population. However, there are many other types of nomads such as hunter-gatherer populations who live in regions where they can rely on non-domesticated plants and animals for food, as well as sea nomads, and traders and craftworkers, who are commonly known as gypsies or Roma [5].

Hundreds of nomadic and pastoralist populations exist all over the world. The exact number of groups and the total population of nomadic people is very difficult to estimate, given their existence at the cultural and geographical periphery of the mainstream societies. The most recent estimate put the population of pastoralists at 30–40 million in the 1990s [5]. The population numbers are largely unknown, and the lack of recent and comprehensive data on global nomadic populations highlights the need for more research and investment in these populations.

Though the cultures and traditions of these groups vary greatly, they are similar in their lifestyles and lack of access to modern health resources. Nomads have historically had low uptake of healthcare services due to many suspected factors such as geographic isolation as well as social disparities [6]. The rate of uptake of reproductive health services among nomadic populations in several African countries is significantly lower than that of the general population [6] . Therefore, reaching the millions of nomads in these underserved communities presents an opportunity for improving global health outcomes and moving toward the SDGs.

Reaching marginalized populations, such as nomads, and better understanding their reproductive health needs has been a focus of WHO [7, 8]. The 2014 guidance on *Ensuring Human rights in the provision of contraceptive information and services* recommends special attention be given to marginalized segments of the population to ensure non-discrimination in the provision of RH services, including family planning information [7]. For populations such as those who have limited access to and low uptake of health services, particular focus must be given to identify innovative approaches to improve access and quality of health services.

This scoping review identifies barriers to access to reproductive health services among nomadic populations from existing literature, and highlights opportunities to increase service provision and uptake among this segment of the population. It specifically responds to the need for ensuring that research and global health focus on populations that are currently underserved in terms of access to information, services, and supplies [8].

## Methods

A scoping review was conducted to identify and analyze relevant literature on barriers to RH service access faced by nomadic populations and to identify possible facilitators to address them. This mapping methodology was chosen as the aim is to summarize existing evidence rather than to assess the quality of the studies themselves [9]. This choice is based on the assumption that the topic has not been researched sufficiently. Additionally, the aim is to understand the contexts in which nomads live and the socio-cultural factors that affect their access to services, which are more often captured in observational studies and reviews rather than experimental designs.

**Fig. 1:**
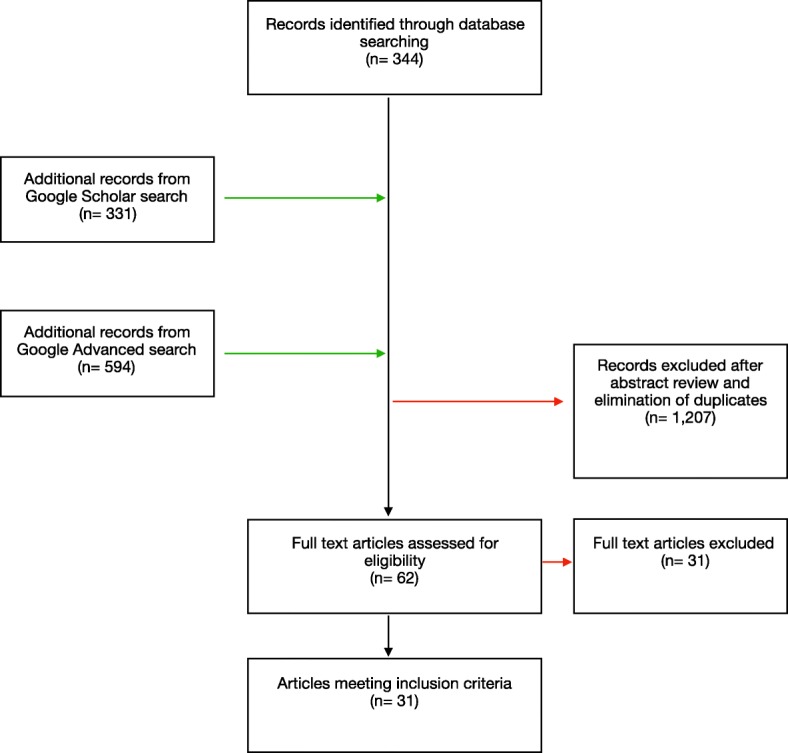
Prisma flow chart showing the different phases of the scoping review

A search strategy was developed using terms based on the themes of “nomadic populations” and “reproductive health services” (Table 1). Pubmed and Popline databases were searched, as well as Google Scholar, to find relevant published literature. Additionally, a grey literature search was conducted using Google Advanced. A first round of search was conducted up to 31 October 2013 and was re-done in February 2017 and re-run again in February 2019 to update the search to January 2019. The results reported here include all rounds of the review: 212 results were found from PubMed, 132 results from Popline, 594 results from Google Advanced and 331 results from Google Scholar. Following abstract and full-text review, 31 articles were included in the review. We have included Prisma flow chart showing the different phases of the scoping review (See Fig. 1 for the Prisma flowchart).

**Table 1:** Search strategy used

Theme	Search words
Nomadic populations	“internal migrants” OR “Bedouin people” OR “nomadic population” OR “nomad people” OR “nomad” OR “bedouins” OR “African nomads” OR “desert nomads” OR “nomads” OR “nomadic people” OR “nomad women” OR “nomad girls” OR “nomads” OR “invisible population” OR “Fulani nomads” OR “Somalia nomads” OR “transients and migrants” OR (“transients” AND “migrants”) OR “transients and migrants” OR “nomads”[All Fields] OR (“African nomads” OR “African nomads”) OR (“Asian nomads” OR “Asian nomads”[All Fields] OR (“middle east nomads” OR (“middle” AND “east” AND “nomads”) OR “middle east nomads”)
AND	
Reproductive health services	“Health Service, Reproductive” OR “Reproductive health services” OR “Health Services, Reproductive” OR “Reproductive Health Service” OR “Service, Reproductive Health” OR “Services, Reproductive Health” OR “Maternal health” OR Matern* OR “Woman Health” OR “Women Health” OR “Woman’s health services” OR “Women Health Service” OR “Women Health Services” OR “Maternal Health Services” OR “Maternal Health Service” OR “Maternal Mortality Rate” OR “Maternal mortality rates” OR “MMR” OR “Female health” OR “Maternal and Child health” OR “Mother and child health” OR “Female and children health” OR “Female and child health” OR “Population control” OR “Population control plans” OR “population control policies” OR “Health care systems” OR “health care facilities” OR “health care facility” OR “Health care seeking behavior” OR “Health seeking behavior” OR “health service utilization” OR “Reproductive behavior” OR “reproductive technologies” OR “High Fertility Population” OR “Maternal-Child Health Services”

All study designs were included as well as published and grey literature that focused on analyzing RH access of nomadic populations. For this review, we define nomadic populations as groups of people whose way of life is defined by not having permanent places of abode and migrates in a seasonal manner [10]. Most of these groups are pastoral nomads in developing countries who do not practice agriculture but rear livestock for their own subsistence or bartering. We also included studies on semi-nomadic groups who engage in mixed form of subsistence involving herding and farming according to the season. Reproductive health services were considered to include provision of family planning and contraceptive services and information and maternal and newborn healthcare services. Additionally, studies included in the review were not restricted by country of origin or language.

We excluded manuscripts that described access to reproductive health services of internal migrants who are not historically nomadic or pastoral groups. We also excluded manuscripts describing urban, peripatetic nomadic groups such as “gypsy” or “Roma” populations.

Data charting was done iteratively. After initial reading of the manuscripts, we identified key themes that relate to access either as facilitators or barriers. Following the identification of the themes, they were grouped into external and internal factors. These themes were used to develop a data-charting template that also included meta-data about the manuscripts (study type, publication status, study design, study site, population, and outcomes measured). Once the data-charting template was established, data were extracted from the manuscripts. When new themes were identified, these were noted and discussed by the authors. When required, the data charting table was updated, and data extraction was conducted again.

Data charting was done iteratively. After initial reading of the manuscripts, we identified key themes to construct a thematic framework (see Table 1). The results of the numerical and thematic analysis are reported in this manuscript.

## Results

### Features of reviewed literature

The 31 articles included in the review were published between 2002 and 2018, with 22 of them being published in peer-reviewed journals [11–32]. Ten articles reported on original research using qualitative methods [11, 12, 15, 16, 20, 24–26, 29, 31], 6 used mixed methods [13, 21, 27, 28, 30, 32], one was based on secondary analysis of data [14], one involved a case study [19], and one used a checklist and standardized SES scores that were developed as part of the study [18]. There were also two review articles [22, 23], one unpublished research that used mixed methods [33], three commentaries [34–36], four reports [6, 37–39], and one thesis for a Master’s degree [40]. Summary of the articles included in this review is provided in Table 2.

**Table 2:** Summary of articles included in the review

Author	Year	Title	Type of publication	Data collection	Nomadic population described
Ahmed, et al.	2018	Sociocultural Determinants of Nomadic Women’s Utilization of Assisted Childbirth in Gossi, Mali: A Qualitative Study	Peer reviewed article: research	Literature review, semi-structured interviews, non-participant observation	Tamasheq (Tuareg) and Fulani in Gossi region of Mali
Sharma et al.	2014	Exploring Tribal Women’s health seeking behaviour in context of demographic and self-related variables	Peer reviewed article: research	Self-devised checklist and standardized SES scale	Gujjars from Jammu Province, India
Assefa, et al.	2018	Magnitude of Institutional Delivery Service Utilization and Associated Factors among Women in Pastoral Community of Awash Fentale District Afar Regional State, Ethiopia	Peer reviewed article: research	Community based cross-sectional study	Pastoral community of Awash Fentale district of Ethiopia
Byrne et al.	2016	Community and provider perceptions of traditional and skilled birth attendants providing maternal health care for pastoralist communities in Kenya: a qualitative study	Peer reviewed article: research	Focus group discussions and interviews with key informants	Pastoralists including Massai in Laikipia and Samburu counties, Kenya
Caulfield et al.	2016	Factors influencing place of delivery for pastoralist women in Kenya: a qualitative study	Peer reviewed article: research	Interviews with key informants and nomad women	Pastoralists including Massai in Laikipia and Samburu counties, Kenya
El Shiekh, et al.	2015	Factors influencing the utilization of maternal health care services by nomads in Sudan.	Peer reviewed article: review	Literature review of published and unpublished studies, research and reports	Nomadic communities in Sudan and neighbouring countries.
Ernest, et al.	2011	Promoting modern family planning among Tanzania’s nomadic communities	Study report	Mixed method research: questionnaire, observation, FGD and IDI	Nomads from Kilindi District in the Tanga region, Tanzania
Gitimu, et al.	2011	Using safe spaces and social networks to convey reproductive health information to nomadic girls	Commentary	Not reported	Maasai from Magadi, Loitokitok in Kajiado County, Kenya
Gyaltsen, et al.	2014	Reducing high maternal mortality rates in western China: a novel approach.	Peer reviewed article: research	Case study	Nomadic herders from the Qinghai province, China
Hampshire	2002	Networks of nomads: negotiating access to health resources among pastoralist women in Chad	Peer reviewed article: research	Qualitative research, including individual interviews, retrospective reporting	Pastoralists from Chad (camel herders near Dourbali; cattle herders near Birbaka; semi-sedentralised Fulani in Kas-samre north of Dougia)
Ibrhim et al.	2018	Reasons for Low Level of Skilled Birth Attendance in Afar Pastoralist Community, North East Ethiopia: A Qualitative Exploration	Peer reviewed article: research	Qualitative methods including focus group discussions and key informant interviews	Pastoralist community in Afar region of Ethiopia
Jackson et al.	2017	Factors That Hinder or Enable Maternal Health Strategies to Reduce Delays in Rural and Pastoralist Areas in Ethiopia	Peer reviewed article: research	Qualitative methods including key informant interviews with community based health extension workers	Pastoralist community in Afar Region, Kafa Zone, and Adwa Woreda (Tigray Region) of Ethiopia
Kazi et al.	2017	Assessing Mobile Phone Access and Perceptions for Texting-Based Mhealth Interventions among Expectant Mothers and Child Caregivers in Remote Regions of Northern Kenya: A Survey-Based Descriptive Study	Peer reviewed article: research	Analysis of surveys given to patients at government health facilities	Pastoralists from Samburu, Isiolo, and Marsabit counties in Northern Kenya
Kruger et al	2011	Where do women give birth in rural Tanzania?	Peer reviewed article: research	Secondary analysis of data from retrospective study	Datoga tribe (nomadic pastoralist) from Northern Tanzania
Le, et al.	2009	Preferences for Perinatal Health Communication of Women in Rural Tibet.	Peer reviewed article: research	Mixed method research: FGD, IDI, 148 women were surveyed	Nomadic herders from Medrogongkar County, Tibet
Lemaron	2016	Community And Institutional Factors Influencing Access To Antenatal Health Care Services By Maasai Women In Isinya, Kajiado County	Master thesis: research	Mixed method research with quantitative and qualitative research features.	Maasai women in Isinya, Kajiado County in Kenya
Lindskog	2014	Natural calamities and ‘the Big Migration’: challenges to the Mongolian health system in ‘the Age of the Market’	Peer reviewed article: research	Ethnographic research	Mobile herders from province of Arhanga, Mongolia
Mansour	2011	Gender at the Margins: Bedouin Women’s Perceptions of Lebanese Health Provision	Peer reviewed article: research	IDI	Bedouin women in Kab Elias and Bar Elias in Middle Bekaa, Lebanon
Mansour, et al.	2014	They aren’t all first cousins: Bedouin marriage and health policies in Lebanon	Peer reviewed article: research	Socioeconomic questionnaires, IDIs with Beduoin women, semi-structured interviews with policy makers	Bedouin women in the Bekaa Valley, Lebanon
Maro, et al.	2012	Myth and Believes associated with Utilization of Family Planning and Maternal health services In Nomadic communities, Kilindi, Tanga Tanzania	Unpublished article: research	Mixed method research: quantitative survey, FGD with young people, IDI	Pure and mixed nomadic communities in the Kilindi District, Tanzania (Nguu, Zigua, Masai, Kaguru Kamba)
Moucheraud et al.	2018	Maternal Health Behaviors and Outcomes in a Nomadic Tibetan Population	Peer reviewed article: research	Secondary analysis of data from surveys	Tibetan nomadic women in Qinghai Province, China
Nduba, et al.	2011	Reproductive health in nomadic communities: challenges of culture and choice	Commentary	qualitative studies (baseline study of AMREF programme)	Nomadic populations in Ethiopia, Kenya and Tanzania
Okeilbunor, et al.	2013	Prospects of using community directed intervention strategy in delivering health services among Fulani Nomads in Enugu State, Nigeria	Peer reviewed article: research	FGDs, IDI among members of nomadic camps and with health providers and programme officers	Nomadic population in the Enugu State, Nigeria
Pettitt	2011	Sexual and reproductive health challenges among Botswana’s San women	Commentary	Not reported	San or Basarwa or Bushmen (hunter-gatherers), Botswana
Sachdev	2012	Perspectives on Health, Health Needs and Health Care Services among Select Nomad Tribal Populations of Rajasthan, India	Peer reviewed article: research	Semi-structured questionnaires	Nomadic tribes in Rajasthan, India (Gadoliya Lohars, Banjaras, Rabaris, Nayaks, Kanjars, Sansis, Nats and Kalbeliyas)
Schelling	2008	Learning from the delivery of social services to pastoralists: Elements of good practice	Report	Literature review	Pastoralists from Western Europe, Latin America, Central, Western and Southern Asia and throughout Africa
Sorbye	2009	Somalia: A situation analysis of reproductive health	Report	Literature review and interviews with implementing actors	Nomads from Somalia
Treister-Goltzman	2014	Health and morbidity among Bedouin women in southern Israel: a descriptive literature review of the past two decades	Peer reviewed article: review	Literature review	Bedouin women in the Negev region of southern Israel
van der Kwaak (ed)	2012	Understanding nomadic realities: case studies on sexual and reproductive health and rights in Eastern Africa	Project report	Case studies from African Medical and Research Foundation (AMREF) Project	Nomadic peoples in East Africa (Maasai of Kajiado and Magadi districts, Kenya; nomadic communities of Kilindi, Tanga, Tanzania; pastoral communities in the Afar Region, Ethiopia)
Wako et al.	2017	Institutional Delivery Service Utilization and Associated Factors among Women of Reproductive Age in the Mobile Pastoral Community of the Liban District in Guji Zone, Oromia, Southern Ethiopia: A Cross Sectional Study	Peer reviewed article: research	Community based cross-sectional survey	Pastoralist community of the Liban District in Ethiopia
Wiese	2004	Participatory mapping as a tool for public health decision-making in nomadic settings. A case study among Dazagada pastoralists of the Bahr-el-Ghazal region in Chad	Peer reviewed article: research	Participatory mapping, FGD and semi-structured interview	Dazagada pastoralists from Bahr-el-Ghazal region, Chad

Much of the literature reviewed in this paper describes nomadic peoples in Africa [11, 12, 14, 17, 23–31, 33–36, 38–42]. Six publications explored sexual and reproductive health access among nomads in Asia, including the different nomadic tribes in India [16, 18] and nomadic herders in China [19], Mongolia [20], and Tibet [13, 32]. Three publications described the Bedouins from Lebanon [15, 21] and Israel [22]. One publication reported on different groups from Western Europe, Latin America, Central, Western, and Southern Asia and throughout Africa [37].

Despite the diversity of the communities reviewed, the studies reported similar poor reproductive health outcomes, including high rates of maternal morbidity and mortality, poor utilization of RH services including modern contraceptive services [4, 6, 13, 15–18, 21–23, 25, 26, 30–32, 35, 38, 40, 42].

### Barriers to accessing reproductive health services

Nomadic populations generally face a wide range of complex barriers that prevent or limit them from accessing reproductive heath services. These barriers can be broadly grouped as external and internal factors. In this paper, we discuss the two categories separately; however, it is important to note that these factors are all inter-related and never exist in isolation. Following the discussion of barriers, we list opportunities gathered from the reviewed literature that should be further investigated and potentially leveraged to increase access to and utilization of RH services for nomadic populations.

#### External factors

Several factors that impede access to RH services among nomadic populations are outside the control of their communities. These include lack of available resources in rural areas and poor quality of services in these remote regions. Nomadic groups also often face added barriers to accessing RH services due to their marginalized status, and in most cases they have little political power to improve their situation.

##### Geographic isolation

Geographic isolation and mobility were identified in this review as two significant barriers to RH health access for nomads. As health facilities are concentrated in more densely populated urban regions, rural areas where most of the nomadic groups are based, have relatively sparse facilities. The long distances that must be traveled to access modern healthcare, often compounded by limited access to modern forms of transportation, make accessing health facilities nearly impossible for many nomads [13, 16, 17, 25, 26, 30, 31, 38]. One study found that pregnant nomadic women in Ethiopia living farther than 30 min from a health center were three times less likely to deliver at a health facility [30]. Numerous interviews with nomads in the reviewed literature revealed that the cost of transportation to health centers is a major barrier, even if the health services provided are free [25, 28, 31]. Not only is it difficult for nomadic peoples to travel to health clinics, it is also difficult and expensive for healthcare workers to travel and bring resources to these groups [37]. The unpredictability and transiency of their physical settlements make it logistically very challenging to provide them with lasting resources and health care services.

##### Quality of health services

Even when services do exist, they are often limited and inefficient due to logistical difficulties [13, 25, 38]. Scarce resources and poor infrastructure in rural health facilities remains a major issue [11, 14, 16, 17, 38, 39]. The quality of reproductive health services suffers from a lack of training for health care workers in isolated rural areas, preventing them from delivering even the most basic services [35]. In instances where a member of a nomadic community has a negative experience or outcome at a rural health facility, their whole community may stop using the facility altogether [25, 26]. This case highlights the importance of providing high quality, consistent care to strengthen relationships between communities and health facilities.

##### Marginalization/Discrimination

Health systems are often designed to serve static populations, with services that cater for nomadic lifestyles rarely being prioritized or even considered by governments [6]. Further, as many nomadic populations are ethnic minorities, RH needs, and practices specific to these populations are often not well understood or are inadequately addressed [21, 31, 35–37]. For example, members of one community of nomads in Ethiopia were reluctant to give birth at the nearby health facility as the health workers refused to provide a suture for complications arising from a delivery that occurred at home [31].

Discrimination against nomadic populations is also a barrier to care. Health officials interviewed in Nigeria considered the Fulani, a nomadic tribe, to be outsiders who take advantage of the resources available to the host community, and are thus reluctant to provide services [17]. Bedouin women in Lebanon perceive the health system as being underpinned by institutional discrimination against their ethnic group [15]. Similarly, surveys and interviews with nomadic women in Kenya, Mali, and India reported mistreatment or discrimination by healthcare workers [16, 25, 26, 29].

##### Vulnerability to political factors

There is often a lack of representation of nomadic cultures in national governments. With a lack of political advocacy, nomadic populations are left out of health policy agendas including those that aim to increase access to modern RH services [15]. Another way in which nomads are vulnerable to political factors is through regional political conflict [29, 38]. In several cases, regions inhabited by nomads are riddled with conflict. This further contributes to a lack of governmental ability to provide adequate healthcare services. One example is Puntland, an independent state within Somalia where the majority of the nomadic population, along with internally displaced people live in a volatile zone with high incidence of conflict [38]. Another example is in Mali, where reproductive health outcomes, such as skilled attendance at birth and maternal mortality have declined since conflict broke out in the regions where nomadic populations live [29].

#### Internal factors

While external factors represent significant barriers to the accessibility of healthcare, internal factors such as cultural, socio-economic barriers can be even more enduring [11]. Low demand for RH among nomadic cultures is a major challenge to providing services to these communities [16, 34, 35]. However, several of the papers reviewed emphasized the acceptability of modern healthcare and their positive impact on the lives of nomads, and a willingness to engage with modern health facilities is possible [17, 26, 29]. Though very challenging, health service delivery can be facilitated through the existing social structures and networks, and the tightly woven communities and strong traditions of these groups can be largely preserved while providing them with improved access to RH services [6, 17, 29, 35].

##### Poverty

Because the nomadic lifestyle is defined by constant movement from place to place depending on livelihood, seasonal changes and availability of resources, most nomadic families have very limited financial resources to spend on healthcare [25, 26, 28, 32]. In some regions, some services such as obstetrical care are provided at no cost, and there is evidence that the uptake of health services has increased as the cost of healthcare goes down [32]. However, the cost of transportation, medicines, and food remain a huge barrier in spite of provision of free health care to many nomadic populations [25, 26, 28, 31]. A study in Ethiopia found that if there was cash readily available for transportation in the homes of nomadic women in labor, they were three times more likely to deliver in a health facility [28]. Compounding this problem is the fact that nomadic women generally do not have financial autonomy, as their work caring for children and family members does not generate income [31]. Therefore, they must rely solely on male family members to provide money to access healthcare.

##### Gender roles

Nomadic women’s ability to make decisions concerning health issues, as well as their autonomy to travel freely to health facilities is often very limited. In most nomadic societies, a woman must seek permission from her husband to access modern health care, including reproductive health services [11, 26, 29, 36]. Even in a situation where a woman is giving birth and needs emergency lifesaving modern healthcare, it is necessary to seek the permission of the husband in some cultures, or if the husband is not present, community leaders must confer and grant permission [29]. This can be a major barrier to accessing RH services, as some husbands and communities can be very resistant to services such as costly delivery at a health center or modern family planning methods [39].

Lack of autonomy of movement for women is another barrier, as nomadic women, especially pregnant women seeking reproductive healthcare, do not travel alone. This limits the accessibility of distant health centers, as male chaperones are often busy with work and unable to accompany women on these long trips. In interviews with nomadic women about their experiences giving birth, many stories outlined situations in which the woman wanted to give birth at a health center, but when the time came to deliver, neither her husband nor other men from the community were present, and she was not able to travel [25, 26, 29–31].

##### Socio-cultural norms

There are several socio-cultural characteristics of nomadic communities that contribute to their lack of uptake of reproductive health services [25, 26, 29, 31]. One strong factor is a deep sense of tradition in many of these communities. In societies whose traditions and autonomous communities have kept them alive in the harshest of conditions over centuries, the tie to these practices can be very strong [25, 26, 29, 31]. Many nomadic women interviewed in the reviewed texts expressed the view that their parents and ancestors had not used modern reproductive health services, “and so we followed in their footsteps” [25, 26, 29, 31].

Another contributing factor to low uptake of RH services is the belief in some nomadic cultures that seeking healthcare or showing illness are signs of weakness [25, 29]. Women may try to hide pregnancies as long as possible, as not to be treated as weak and fragile [29]. Talking about RH issues and sex is taboo in many cultures as well, which can decrease the demand for reproductive health services [29]. Additionally, in many nomadic cultures, a high fertility rate is regarded as positive and is often associated with social standing [35]. Thus, contraceptive and family planning methods are not commonly sought.

Several nomadic groups also practice traditions that may be harmful to reproductive and sexual health of women and girls. In some nomadic cultures in Ethiopia, Kenya, Somalia, and Tanzania female genital mutilation (FGM) remains common practice [6, 25, 35, 38]. The practice persists despite strong international campaigns to eliminate it and evidence showing that it can have adverse effects and may lead to difficulties during childbirth [35].

##### Perceptions and attitudes toward modern reproductive health

Among nomadic communities, a lack of awareness about modern reproductive health services and their benefits is a barrier to utilization [15, 38]. In some cases, even where there is a general understanding of RH issues, the understanding may be basic and contain misconceptions. Misconceptions about medical procedures and side effects can produce a negative impression of modern healthcare in communities.

For example, in a nomadic community in Ethiopia, pregnant women are reluctant to visit health centers as they are fearful of having a cesarean section and do not understand the possible benefits of the procedure [26]. Another example of this problem is shown in results of a programme implemented by the African Medical Research Foundation (AMREF) which found that although knowledge of HIV/AIDS among nomadic youths exists, it is often very basic [35]. The study also found that young nomads in Ethiopia did not consider themselves to be at risk of sexually transmitted diseases, and therefore were unlikely to pursue testing [35]. Similarly, 77% of youths in nomadic groups in Tanzania reported knowledge of at least one modern method for avoiding pregnancy but chose not to use them due to misconceptions concerning side-effects [39]. In addition, Fulani nomadic women expressed a reluctance to utilize modern health care facilities due to cultural constraints and religious beliefs, even in the case where the health center was only three to four kilometers away [11].

In many nomadic communities, traditional herbalists, healers or traditional birth attendants (TBAs) are perceived to be more reliable sources of information and health services compared to formal healthcare workers. They are seen as being culturally closer to the people, trusted, and very knowledgeable on community health problems [24–26, 35]. In the case of childbirth, home deliveries are preferred and are generally overseen by traditional healers and midwives who are usually older women with no formal training [11, 24–26, 34].

### Opportunities

A number of strategies to address these barriers and improve the RH of nomadic populations have been outlined in the literature reviewed. Targets for intervention range from governmental policy to the workings of rural health facilities, and also emphasize the importance of addressing cultural practices among the nomadic populations.

#### Improvement of facilities and healthcare system

Reproductive health facilities that serve these populations could be expanded and improved to provide higher quality care. This can be achieved through increased investment from governmental, non-governmental and international bodies. The needs of these underserved populations should be addressed in national policy frameworks and community-based campaigns for improving reproductive and sexual health [36]. There are several cases where the poor quality of health facilities negatively impacted the trust of nomadic communities in modern medicine [25, 26, 31].

Strengthening healthcare systems, and working towards the goal of universal health coverage would also benefit nomadic populations. As discussed above, the financial burden of healthcare represents a significant barrier to access for many nomads. Ameliorating this burden with healthcare coverage can greatly improve access for nomads in particular and impoverished communities in general [32].

#### Mobile health care services or outposts

Decentralization of health care services through provision of mobile services can be an important step to ensuring effective delivery of RH services including family planning tools, maternal health monitoring and neonatal and child health [35, 39]. One study examined examples of successful implementation of mobile health services that target nomads. The examples included the mobile healthcare system by AMREF and the Kenyan Ministry of Health’s steps towards addressing the needs of the Maasai [35]. This specific intervention consisted of a mobile health clinic that provided essential drugs and services for treating common medical problems that moved between settlements in pastoral zones of the country for 2 weeks at a time. Employees in the clinic were able to address basic healthcare needs and referred patients with more severe problems to the nearest fixed health clinics. The study similarly demonstrated the efficiency of delivering healthcare services when combining both mobile and fixed services in Niger [37]. However, one major barrier to this program was the immense cost required to travel to the nomadic populations, especially considering that the mobility of the populations made their locations largely unpredictable [37].

#### Increase in the healthcare workforce

In some regions, a lack of trained healthcare providers inhibits nomadic populations from getting adequate healthcare [26]. A Health Extension Worker (HEW) program is one of the initiatives implemented by the Ethiopian government to educate, train and support community-based healthcare workers who can then serve as liaisons between nomadic populations and the healthcare system in the country. These HEWs are also trained along with traditional birth attendants to provide assistance during home-based child delivery [26]. The uptake of HEW and trained TBA services among nomads in Ethiopia has been promising, and interviews reveal that it is easier for women to seek care from within their community compared to distant health facilities [26]. However, there are not enough HEWs to adequately meet the needs of the broadly scattered nomadic populations, according to an interview with a health bureau representative in Ethiopia [31]. Additionally, TBAs are not trained or meant to deliver babies independently and must work in coordination with skilled birth attendants in these scenarios. There is a clear need not only for more of these community-based health workers but also skilled medical professionals in the areas where nomadic populations reside.

Traditional healers and herbalists play a significant role in providing reproductive health services in nomadic communities. Additionally, nomadic communities often perceive them as more effective and reliable than modern healthcare workers [24–26, 31]. Providing formal training to traditional healers and midwives and helping them integrate modern reproductive healthcare methods has been shown to improve reproductive health outcomes such as maternal mortality [24, 26]. Increased training and education of traditional health providers can also aid in decreasing harmful practices such as FGM [35].

#### Sensitizing health providers toward the needs of nomads

The literature also recognizes the need for culturally sensitive services geared towards nomads and pastoralists. In cases where there is a lack of knowledge about the culture and traditions of the regional nomadic populations, there is potential loss of trust between health facilities and communities [24–26]. On the other hand, when a woman has a positive experience at a health center, for example a smooth childbirth experience with staff who treat her kindly, she will most likely return [29]. Culturally sensitive healthcare workers are in a better position to gain trust and dispel misconceptions in communities surrounding reproductive health services [15]. Participatory tool mapping of nomadic populations can help gain an insight into their beliefs regarding reproductive health and could act as an essential tool to educate nomads about modern family planning methods [12]. Programmes integrating a better understanding of nomadic decision-making patterns and specific cultural practices have also shown great potential [13, 15, 17].

#### Focusing on nomadic youths

There is a huge potential to reach young people in nomadic communities. Changing the perceptions of reproductive healthcare among youths can have long-term impact on the way health services will be utilized. However, in some nomadic communities, young women are less likely than older women to use reproductive health services [29]. The Nomadic Youth Reproductive Health Project (NYRHP), conducted by AMREF in East Africa focused on nomadic youth’s knowledge about RH services. The results of the programme similarly showed that young people have the lowest uptake of RH services. The study concluded that programmes and services directed specifically at youths should be prioritized [6, 34, 35]. One report, which focused on the health needs of Maasai girls, showed that creating safe environments where young women can share their concerns and ask questions about reproductive health is an effective strategy to improve perceptions about the services [34]. There has also been a demonstrated desire for more mHealth resources by pastoralist women in Kenya [27]. Though the study did not specifically survey nomadic youth, the increasing uptake of mobile devices among young people around the globe could open up opportunities to distribute reproductive health information, and link these populations to health care systems.

#### Involving the entire community in reproductive health education

As husbands, parents, and community members are often the gatekeepers to reproductive health services for nomadic women, interventions targeting entire communities as opposed to only women of reproductive age could be better-explored. For example, many women prefer to give birth at health facilities, but when the time comes to deliver they have no plan in place to go to the centers. When women discuss their wishes and make plans with their husbands well ahead of time, they are more likely to deliver at facilities with skilled healthcare workers present [30, 31]. In one community in Ethiopia, after poor outcomes where no men were available to accompany women in labor with obstetric complications to health facilities, the community decided that several men would always be on call to stop working and travel with pregnant women if needed [26]. Similarly, successful strategies to improve the reproductive health of Maasai women and girls involved bringing young warriors on board, as they are seen as the custodians of culture [34].

A survey of women living in rural Tibet showed that pregnant women were much more likely to take advice and counseling from close relatives as opposed to health workers or media [13]. This reiterates the importance of educating entire communities on the benefits of modern reproductive health services to reach women of reproductive age. Community leaders can also increase the impact of community health workers such as the HEWs in Ethiopia. When HEWs are supported and trusted by community members, they can provide high quality reproductive health care [6].

#### Improving education for girls

Improving and increasing education for girls and women is key to improving access to reproductive health services for nomads [25, 35]. A study of nomadic women in Tibet revealed that the more educated women were more likely to seek modern reproductive healthcare [32]. The ability to read can increase a woman’s access to health materials in innumerable ways by enabling her to utilize written information about healthcare. One study found that while a large proportion of nomadic families have access to mobile devices in northern Kenya, relatively few women can read, and therefore were more limited in their ability to use the devices to access health information [27]. With improved literacy, the increasing prevalence of mobile devices can be leveraged in many ways to provide reproductive health information to nomadic women [27, 34]. Increased education for girls can also improve social conditions in communities in general and have positive effects on access to reproductive health. Improved school attendance among nomadic youth and availability of income-generating activities for nomadic women increase autonomy and are among the recommended strategies to improve reproductive health for nomads [25, 34, 35].

## Discussion

Progress toward Universal Health Coverage (UHC), which means that all individuals and communities receive the health services they need without suffering financial hardship, can help countries move forward towards achieving their health-related SDGs [43]. This includes SDG 7 which focuses on ensuring universal access to sexual and reproductive health-care services, including family planning, information and education, and the integration of reproductive health into national strategies and programmes. UHC is not just about health but also focuses on taking steps towards equity, development priorities, and social inclusion and cohesion.

Nomads are amongst the most underserved and hard to reach populations facing increasing challenges [44]. The results of this review may help stakeholders to understand the challenges better and develop a concerted approach to improve the provision of RH services to nomadic populations. It may provide information to adapt proven approaches to target the specific needs of nomadic populations i.e. recruiting nomadic people and training them to be HEW, educating women and girls generally and with specific focus on nomadic women and children and understanding startegies that work with them.

In order to improve the health of these marginalized populations, governments must be willing to invest resources and implement policies to provide better services in rural, nomadic regions. Policy supporting education and promoting positive reproductive health practices while condemning harmful practices such as FGM can also aid in improving the reproductive health outcomes of nomadic populations. For example, interventions to improve the RH of nomadic communities in Kenya have yielded positive outcomes. Another successful example has been the training of traditional birth attendants (TBA) in rural Ethiopia. The literature reviewed showed that trained TBAs have been vital in improving uptake of modern reproductive health services by reaching out to women and counselling them and also referring women for services [43]. Investing in improving TBA programmes is critical, as their contributions can be broadened through improved training, refresher courses, and more formalized referral systems to health facilities [24, 31, 43].

Training community members of nomadic groups to become CHW or HEW has been found to promote equity in access to and utilization of health services by reducing inequities relating to place of residence, gender, education and socio-economic position [45]. Recruiting CHWs from the community and the existing relationships they have with community members contribute to improved uptake of the services they provide and thus improve equity [45].

Special services for nomads must be integrated into the existing healthcare infrastructure in countries to ensure sustainability and that these populations can access the same quality of care as the general population. For instance, mobile health clinics and the use of volunteer healthcare workers have proven to be very effective in reaching nomadic groups in remote rural areas. However, these efforts are expensive, and require significant financial and logistical support to have an impact on populations. They should be recognized as essential means of disseminating healthcare and be integrated into existing health systems. There is need to engage healthcare providers serving nomadic populations in the design, implementation and scaling up of effective and sustainable interventions.

The review also highlights the need for investment in education for women and girls from nomadic population globally. Several of the studies reviewed showed that uptake of RH services, and access to mobile health services was higher among educated women. Education is important for autonomy and independence of nomadic women regarding decisions to use healthcare services. With improved education, improved resources, and more independence, women are more likely to seek healthcare and use RH services. However, improving formal education of girls from nomadic populations is difficult due to the transient nature of these groups, the strong traditional societies where they reside, and the sometimes antagonist relationships these groups have with the government in the countries in which they reside. The structure and rigidity of schedules in formal schools make it difficult for nomadic children to regularly attend lessons. Although it is not the purpose of this review to outline education systems and strategies that are appropriate for nomadic populations, it is important to draw attention to the fact that healthcare uptake is intimately intertwined with education systems globally, and investing time and resources in education for girls can have significant impacts on the health and wellbeing of communities.

This review reveals that there are many opportunities to engage with nomadic communities to improve access to reproductive health services and ensure culturally appropriate interventions. Nomadic community members should be actively involved in the planning and implementation of the health programmes to ensure that their needs and concerns are addressed. Community involvement will also ensure that the programmes are effectively implemented and can leverage the existing structures, networks and decision-making patterns in the community. Engaging male partners and male community leaders have the potential to increase service utilization as they have decision-making powers [31]. Interventions that also aim to engage and improve men’s knowledge and understanding of sexual and reproductive health can address poor knowledge and socio-cultural barriers [41].

This review aimed to elucidate barriers to accessing care and to explore opportunities to improve uptake of reproductive health services among nomadic populations. Limitations to this study include the fact that this was a broad scoping review, which may have missed some possible barriers and opportunities to uptake of reproductive health services among nomadic populations. There is immense diversity among nomadic people globally, and grouping together small communities from different continents and regions may have led us to make generalizations that may not necessarily apply to specific nomadic populations. The barriers and opportunities identified in this review may not apply to many communities that were not studied in the reviewed literature. Many of the studies in our review were primarily qualitative and consisted largely of interviews with nomads and healthcare providers. While this helped achieve the goal of identifying real-world problems faced by these people, the number of documents considered is limited. The limited number of documents is also due to the paucity of research on the topic. These limitations highlight the need for further research on the topic involving various study designs for data collection as well as monitoring and evaluation of programmes to improve uptake of services among this segment of the population.

Whereas there are efforts to provide services to these hard-to-reach populations, they are sometimes hampered by lack of evidence to inform effective implementation as well as limited understanding of nomads and their culture [34]. A clear understanding of the unique needs and diversity of nomads is often lost in large-scale studies that focus on broader population-based issues.

## Conclusion

One of the pillars of the 2030 Agenda for Sustainable Development and the Sustainable Development Goals is the pledge to ‘leave no one behind.’

Research exploring sociocultural and structural factors as well as understanding the needs of nomads can play a critical role in developing health services. Additionally, implementation or operations research that aims to understand how well proven interventions perform when adapted to nomadic groups is needed.

There appears to be increasing efforts to understand the specific contexts and barriers faced by nomadic people in accessing reproductive health services. Although the cultures, traditions, and contexts in which nomadic groups live are very diverse, they face similar barriers and opportunities which programmes and policies should consider.

## Data Availability

It is a review article, and all the included articles are available online in global databases such as medline/pubmed, etc. If need be, the reviewed articles will be available upon request. Requests can be sent to alimoa@who.int

## Abbreviations

AIDS: Acquired immune deficiency syndrome
AMREF: African Medical Research Foundation
FGM: Female genital mutilation
HEW: Health Extension Worker
HIV: Human immunodeficiency virus
NYRHP: Nomadic Youth Reproductive Health Project
RH: Reproductive health
RH: Reproductive Health
SDG: Sustainable Development Goal
SES: Socioeconomic status
TBA: Traditional birth attendants
WHO: World Health Organization
